# NK cells in human visceral adipose tissue contribute to obesity‐associated insulin resistance through low‐grade inflammation

**DOI:** 10.1002/ctm2.192

**Published:** 2020-10-06

**Authors:** Kristiaan Wouters, Yvo H.A.M. Kusters, Mitchell Bijnen, Suzan Wetzels, Xiaodi Zhang, Pauline B.C. Linssen, Katrien Gaens, Alfons J.H.M. Houben, Peter J. Joris, Jogchum Plat, M. Eline Kooi, Carla J.H. van der Kallen, Ronald P. Mensink, Kenneth Verboven, Johan Jocken, Dominique Hansen, Ellen E. Blaak, Femke A.I. Ehlers, Lotte Wieten, Jan Willem Greve, Sander Rensen, Coen D.A. Stehouwer, Casper G. Schalkwijk

**Affiliations:** ^1^ Departments of Internal Medicine MUMC+ Maastricht The Netherlands; ^2^ Human Biology (NUTRIM) MUMC+ Maastricht The Netherlands; ^3^ Nutrition and Movement Sciences (NUTRIM) MUMC+ Maastricht The Netherlands; ^4^ Radiology and Nuclear Medicine MUMC+ Maastricht The Netherlands; ^5^ CARIM School for Cardiovascular Diseases MUMC+ Maastricht The Netherlands; ^6^ Top Institute Food and Nutrition Wageningen The Netherlands; ^7^ Department of Immunology and Biochemistry Biomedical Research Institute (BIOMED) Faculty of Rehabilitation Sciences Hasselt University Diepenbeek Belgium; ^8^ Rehabilitation Research Center (REVAL) Biomedical Research Institute (BIOMED) Faculty of Rehabilitation Sciences Hasselt University Diepenbeek Belgium; ^9^ Heart Centre Hasselt Jessa Hospital Hasselt Belgium; ^10^ GROW School for Oncology and Developmental Biology Maastricht University Maastricht Netherlands; ^11^ Tissue Typing Laboratory Department of Transplantation Immunology MUMC+ Maastricht Netherlands; ^12^ Department of Surgery (NUTRIM) MUMC+ The Netherlands; ^13^ Department of General Surgery Zuyderland Medical Centre Heerlen The Netherlands

Dear Editor,

Obesity leads to macrophage infiltration in adipose tissue (AT), causing chronic low‐grade inflammation (LGI). This in turn leads to insulin resistance, contributing to the increased risk for type 2 diabetes in obese individuals. Obesity shifts the polarization status of adipose tissue macrophages (ATM) away from an anti‐inflammatory or “M2" phenotype toward the inflammatory “M1” state, hallmarked by the surface expression of CD11C and the production of inflammatory cytokines.^1^ The exact mechanisms that trigger ATM accumulation and activation remain to be elucidated. Animal studies identified that natural killer (NK) cells are able to induce M1 ATM accumulation by producing interferon gamma (IFN‐γ), tumor necrosis factor (TNF), or interleukin (IL)‐6, culminating in insulin resistance.^2^, ^3^, ^4^ NK cells function perform surveillance and elimination of virally infected, tumorigenic or stressed cells, also leading to cytokine production.^5^ NK cells were found to accumulate in human adipose tissue of obese individuals,^6^ but whether they contribute to accumulation and polarization of ATMs and insulin resistance in humans remains unknown. We therefore investigated NK cell accumulation in human adipose tissue and their potential contribution to low‐grade inflammation and insulin resistance in humans.

We collected NK cells from lean and obese individuals and cultured them with primary monocyte‐derived macrophages from a healthy donor. Compared to controls, NK cells from obese donors contained elevated levels of TNF. Moreover, they promoted M1 polarization of monocyte‐derived macrophages (Figure 1A,B; Figure S1A‐D). These results are in line with results showing enhanced TNF production by NK cells in epididymal AT from obese mice.^2^ Although data in mouse models show that also IFN‐γ produced by NK cells causes inflammation and insulin resistance,^4^ we did not observe increased IFN‐γ in NK cells of obese individuals (data not shown).

**FIGURE 1:**
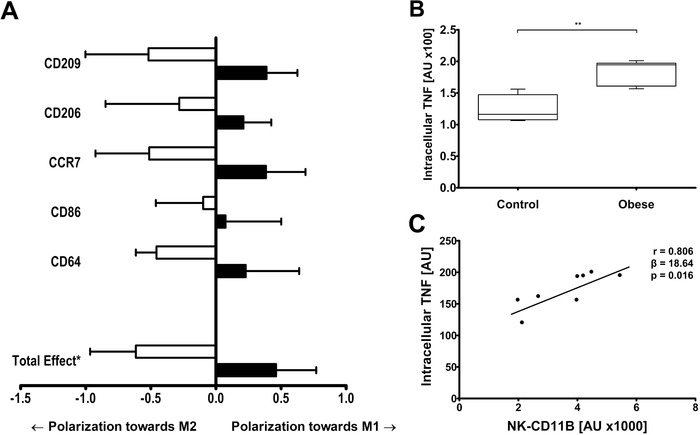
NK cells from obese subjects promote inflammatory macrophage polarization and produce increased levels of TNF. A, Blood‐derived NK cells from control (n = 6; BMI 24.0 [22.5–26.2] kg/m^2^; white bars) and obese individuals (n = 8; BMI 41.5 [40.4–47.0] kg/m^2^; black bars) were isolated and co‐cultured with either human monocyte derived macrophages to analyze surface expression of M1 markers (CCR7, CD86, and CD64), or incubated with macrophages that were first polarized to an M2 phenotype and assessed for loss of M2 markers (loss of CD209 and CD206; that is, polarization toward M1). An aggregate total effect of polarization toward an M1 phenotype was calculated (ie, a *z*‐score composed of the individual *z*‐scores of ‐CD209, ‐CD206, CCR7, CD86, and CD64). Compared to lean individuals, NK cells from obese individuals induced macrophage polarization toward the M1 phenotype (mean difference 1.08 SD [95% CI 0.06–2.10 SD; ^*^ *P* < .05]). Data are presented as mean ± SEM. B, Intracellular TNF levels were measured in blood‐derived NK cells of lean (*n* = 4) and obese individuals (*n* = 6) using flow cytometry. Box plot is as follows: black line, median; box edges, first and third quartiles; whiskers, minimum, and maximum of all data. C, Association between CD11B surface expression and intracellular TNF levels of blood‐derived NK cells (n = 8). Pearson's correlation coefficient, regression coefficient, and their *P*‐value are reported

Then, we analyzed blood, visceral adipose tissue (VAT), and subcutaneous adipose tissue (SAT) from lean and obese men (baseline characteristics in Table S1).^7^ NK cells were higher in VAT (Figure 2A) and blood, but not in SAT from obese subjects (Table S1). In VAT, but not in SAT, NK cell abundance was associated with an increased inflammatory macrophage polarization (Figure 2B). These data support the previously found role for NK cells in skewing M1 macrophage polarization specifically in VAT of mice.^2^, ^3^, ^4^ Previous studies also show that human VAT contains more inflammatory NK cells than SAT^8^ and that VAT contains ligands of the NK cell activating receptor NKp46.^4^ Together with our observations, these data suggest that NK cells can sense stressed VAT adipocytes, accumulate in VAT and TNF production, leading to ATM activation.

**FIGURE 2:**
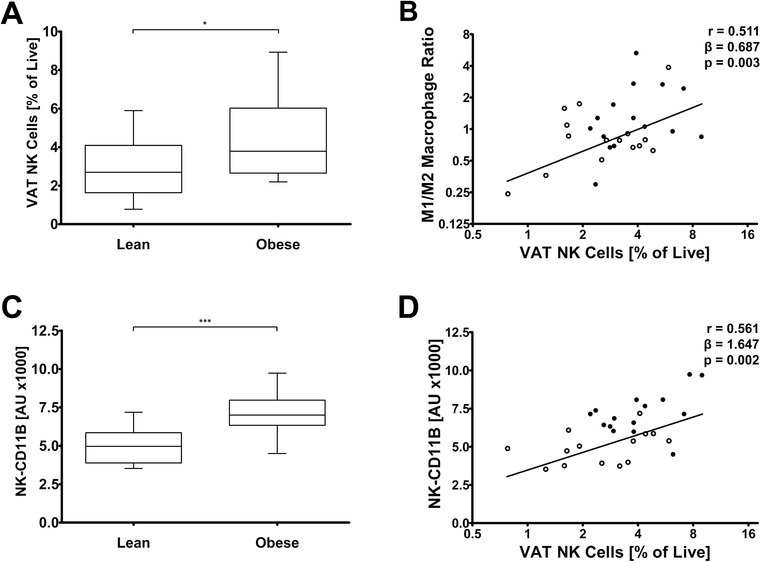
NK cell accumulation in VAT biopsies is increased in obesity, associated with inflammatory macrophage polarization, and reflected by CD11B surface expression on circulating NK cells. A, NK cells in visceral adipose tissue (VAT) in lean versus obese individuals (n = 31; ^*^ *P* < .05). B, Association between NK cells in VAT and M1‐to‐M2 ratio in VAT (n = 31). C, CD11B expression on circulating NK cells (NK‐CD11B) in lean versus obese individuals (n = 31; ^***^ *P* < .001). D, Association between NK cells in VAT and NK‐CD11B (n = 29). Differences between groups were assessed by independent Student's *t*‐tests (panels A and C). Box plots are as follows: black line, median; box edges, first and third quartiles; whiskers, minimum, and maximum of all data. Pearson's correlation coefficients, regression coefficients, and their *P*‐values are reported for all bivariate associations (panels B and D). Data of lean (○ open circles) and obese men (● closed circles) are shown

To obtain biopsies of VAT in large cohort studies is difficult. Therefore, we sought for a marker of the accumulation of NK cells in VAT. Circulating NK cell numbers were not associated with NK cells in VAT (*r* = 0.058, *P* = 0.759). However, surface expression of CD11B on blood NK cells (referred to as NK‐CD11B) was a suitable marker. Despite that all NK cells express CD11B, NK‐CD11B was elevated in obese men (Figure 2C; Figure S1E) and was closely associated with the accumulation of NK cells in VAT (Figure 2D), but not in SAT. In line with our results showing an association between VAT NK cells and macrophage polarization, also M1/M2 ratio was found to associate with NK‐CD11B (*r* = 0.364; *P* = 0.048). Of note, there was also a close association between surface expression of CD11B on isolated NK cells and intracellular TNF (*r* = 0.806, *P* = 0.016; Figure 1C). These important findings support the use of NK‐CD11B as an easy‐to‐measure marker of NK cells in VAT.

We next measured NK‐CD11B to determine the link between VAT NK cells and insulin resistance, using a study consisting of 53 abdominally obese and 25 lean men^9^ (Clinical characteristics: Table S2). Also here, NK‐CD11B was elevated in obese compared to lean men (Figure 3A), thus reflecting increased NK cells in VAT. NK‐CD11B was also associated with VAT volume (Figure 3B) and LGI (Figure 3C). Interestingly, NK‐CD11B was inversely associated with whole body glucose disposal (Figure 3D). Since NK‐CD11B reflects VAT NK cell accumulation, these results corroborate with previously identified findings showing that NK cells cause insulin resistance in mice,^2^, ^3^, ^4^ and that these events are also of importance in humans. Additionally, with multiple mediation analyses we explored if NK‐CD11B and consecutive systemic LGI contribute to the existing association between VAT volume and insulin resistance. NK‐CD11B explained 53.9% of the contribution of LGI to VAT‐associated insulin resistance (Figure 3E). This was mainly mediated by TNF (β –0.048 [–0.170 to –0.006]), but not by IL‐6 (β –0.018 [–0.077 to 0.043]). Together with our data showing that NK‐CD11B and intracellular TNF levels in circulating NK cells were closely associated (Figure 1C), these analyses suggest that NK cell recruitment is an important event that contributes to the development of inflammation in VAT and systemic LGI in human*s*.

**FIGURE 3:**
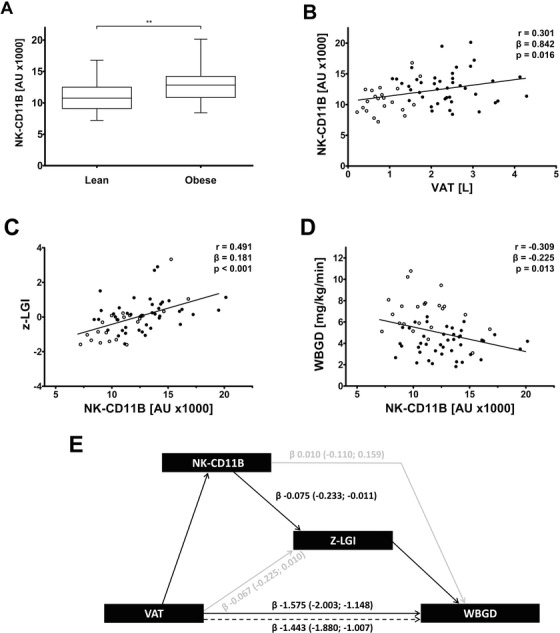
NK cell accumulation is associated with VAT volume, low grade inflammation, and insulin resistance in abdominally obese men. A, CD11B expression on circulating NK cells (NK‐CD11B) in lean versus abdominally obese individuals (n = 64; ^**^ *P* < 0.01). B, Association between visceral adipose tissue volume (VAT) and NK‐CD11B in lean and abdominally obese individuals (n = 63). C, Association between NK‐CD11B and low‐grade inflammation (z‐LGI; composite *z*‐score of CRP, SAA, IL‐6, IL‐8, sICAM‐1, and TNF) in lean and abdominally obese individuals (*n* = 64). D, Association between NK‐CD11B and whole‐body glucose disposal (WBGD) during a hyperinsulinemic, euglycemic clamp in lean and abdominally obese individuals (n = 64). Difference between groups was assessed by means of independent Student's *t*‐test (panel A). Box plots are as follows: black line, median; box edges, first and third quartiles; whiskers, minimum, and maximum of all data. Pearson's correlation coefficients, regression coefficients, and their *P*‐values are reported for all bivariate associations (panels B‐D). Data of lean (○ open circles) and obese men (● closed circles) are shown. E, Multiple mediator model in which CD11B expression on circulating NK cells (NK‐CD11B) statistically significantly contributes through low‐grade inflammation (z‐LGI; composite z‐score of serum CRP, SAA, IL‐6, IL‐8, sICAM‐1, and TNF levels) to the association between abdominal visceral adipose tissue (VAT) and insulin resistance by means of whole‐body glucose disposal (WBGD; n = 63; age‐adjusted). Data presented as β of mediated effect (bootstrapped 95% CI). Significant associations are represented by black lines, whereas grey lines represent associations that are not statistically significant

For the validation of our findings in the general population, we used a population‐based cohort study enriched in individuals with type 2 diabetes, “The Maastricht Study” (Table S3). VAT volume, assessed by MRI, was found to associate with NK‐CD11B (β = 3.2, *P* = .006; Table S4). This result was not affected by further adjustment for SAT volume (not shown). Moreover, NK‐CD11B was associated with LGI and circulating levels of TNF and IL‐6 (*P* < 0.05 for each; Table S5). NK‐CD11B was also associated with insulin resistance (Table S6). These results support the concept that NK cells play a role in low‐grade inflammation and insulin resistance development in the general population.

To conclude, we show that NK cells contribute to inflammatory macrophage polarization, LGI, and concomitant insulin resistance in humans, possibly via TNF production. Thus, targeting NK cells may be promising for the inhibition of insulin resistance and possibly for the prevention of progressing toward type 2 diabetes. Moreover, our study identified that CD11B surface expression on blood NK cells closely reflect NK cell accumulation in VAT, making it an easy to use biomarker of VAT NK cells.

## AUTHOR CONTRIBUTIONS

KW designed, supervised, and performed experiments in all studies, analyzed the data and wrote the manuscript; YHAMK performed experiments in the metabolic phenotyping study and performed the statistical analyses of all studies, analyzed data, and wrote the manuscript; MB and XZ performed experiments and analyzed data; SW performed experiments; PBCL analyzed data of the validation study; KG performed experiments and analyzed data of the first biopsy study; YHAMK, AJHMH, PPJ, JP, RPM, CGS, and CDAS designed the metabolic phenotyping study; EK designed the MRI protocols; CJHvdK and CDAS designed and supervised the validation study (The Maastricht Study); KV collected samples, performed measurements, and recruited individuals undergoing surgery in the first biopsy study; JJ performed experiments in the first biopsy study; DH and EEB supervised experiments in the first biopsy study; FAIE and LW designed and performed the *ex‐vivo* experiments from the second biopsy study; SR and JWG designed and supervised experiments in the second biopsy study; CGS supervised experiments and wrote the manuscript. All authors critically read and commented on the manuscript. None of the authors have a conflict of interest to declare.

## Supporting information

Supporting information

## References

[1] Hill AA , Reid Bolus W , Hasty AH . A decade of progress in adipose tissue macrophage biology. Immunol Rev. 2014;262(1):134‐152. 25319332 10.1111/imr.12216 PMC4203421

[2] Lee BC , Kim MS , Pae M , et al. Adipose natural killer cells regulate adipose tissue macrophages to promote insulin resistance in obesity. Cell Metab. 2016;23(4):685‐698. 27050305 10.1016/j.cmet.2016.03.002 PMC4833527

[3] Theurich S , Tsaousidou E , Hanssen R , et al. IL‐6/Stat3‐dependent induction of a distinct, obesity‐associated NK cell subpopulation deteriorates energy and glucose homeostasis. Cell Metab. 2017;26(1):171‐184 e6. 28683285 10.1016/j.cmet.2017.05.018

[4] Wensveen FM , Jelencic V , Valentic S , et al. NK cells link obesity‐induced adipose stress to inflammation and insulin resistance. Nat Immunol. 2015;16(4):376‐385. 25729921 10.1038/ni.3120

[5] Vivier E , Raulet DH , Moretta A , et al. Innate or adaptive immunity? The example of natural killer cells. Science. 2011;331(6013):44‐49. 21212348 10.1126/science.1198687 PMC3089969

[6] O'Rourke RW , Gaston GD , Meyer KA , White AE , Marks DL . Adipose tissue NK cells manifest an activated phenotype in human obesity. Metabolism. 2013;62(11):1557‐1561. 24012153 10.1016/j.metabol.2013.07.011 PMC3809342

[7] Wouters K , Gaens K , Bijnen M , et al. Circulating classical monocytes are associated with CD11c+ macrophages in human visceral adipose tissue. Sci Rep. 2017;7:42665. 28198418 10.1038/srep42665 PMC5309742

[8] O'Rourke RW , Metcalf MD , White AE , et al. Depot‐specific differences in inflammatory mediators and a role for NK cells and IFN‐gamma in inflammation in human adipose tissue. Int J Obes (Lond). 2009;33(9):978‐990. 19564875 10.1038/ijo.2009.133 PMC3150185

[9] Kusters YH , Schalkwijk CG , Houben AJ , et al. Independent tissue contributors to obesity‐associated insulin resistance. JCI Insight. 2017;2(13). 10.1172/jci.insight.89695 PMC5499371 28679946

