# Supplementary material for: NK cells in human visceral adipose tissue contribute to obesity‐associated insulin resistance through low‐grade inflammation
Source: Clin Transl Med. 2020 Oct 6;10(6):e192. doi: 10.1002/ctm2.192 (PMC7537422; doi:10.1002/ctm2.192)
Supplement: Supplementary file 1 — Supporting information [file CTM2-10-e192-s001.docx]

**Supplementary Materials and methods**

*Biopsy Studies*

In the first biopsy study, age-matched lean (n=15) and obese (n=17) men scheduled to undergo abdominal or bariatric surgery were recruited (1). After an overnight fast, blood was drawn and adipose tissue biopsies were taken from the abdominal subcutaneous adipose tissue depot (SAT) and the omentum majus (VAT) during surgery. Blood and stromal vascular fraction (SVF) cells were obtained by collagenase digestion of adipose tissue and stained for flow cytometry. The study was approved by the medical ethical committee (NCT02598544) and performed in accordance with the Declaration of Helsinki, and all individuals gave their written informed consent before participating in the study.

*Metabolic phenotyping study*

In this cross-sectional study with blinded analyses, 53 abdominally obese and 25 lean men were recruited as described previously (2). Participants had a waist circumference below 94 cm (lean) or between 102-110 cm (abdominally obese). All participants gave written informed consent. The study was approved by the institutional medical ethical committee (NL41397.068.12) and performed in accordance with the Declaration of Helsinki. Metabolic insulin sensitivity was assessed by a 1 mU/kg/min euglycemic insulin clamp as described previously (3). Information on visceral and subcutaneous adipose tissue volumes was obtained through two-dimensional T1-weighted turbo spin echo imaging on a 3.0T Philips Achieva MRI scanner with a dedicated sixteen-element torso coil (XL Torso coil, Philips Healthcare, Best, The Netherlands) as described (2).

*Validation study (The Maastricht Study)*

For this validation dataset we used data from the Maastricht Study, a population-based cohort study. The rationale and methodology have been described previously (4). For this study, fresh whole blood of 1104 participants of the Maastricht Study consecutively enrolled between January and November 2013 were used for flow cytometry. The examinations of each participant were performed within a time window of three months. The study has been approved by the institutional medical ethical committee (NL31329.068.10) and the Minister of Health, Welfare and Sports of the Netherlands (Permit 131088-105234-PG). All individuals gave written informed consent. Magnetic resonance images were acquired using a 3T MRI system (MAGNETOM Prisma^fit^, Siemens Healthineers, Erlangen, Germany) with body matrix and supine radiofrequency coils.

*Flow cytometry*

Fresh heparinized whole blood (50 µl) (biopsy study and Maastricht Study) or thawed whole blood (Metabolic phenotyping study) was used for staining for flow cytometry (antibodies and gating strategy: Supplementary table 7 and Figure S2) (1).

Isolated SVF cells from the first biopsy study were stained as described (1) (antibodies and gating strategy: supplementary table 8 and Fig. S3). Samples were measured with a FACS-Canto II (BD Biosciences). Results were analyzed with FACSdiva software (BD Biosciences). Each panel was optimized using fluorescence minus one controls and isotype controls, yielding similar measures as auto-fluorescent samples. For each panel, an auto-fluorescence control was performed.

*NK cell measurement and isolation.*

PBMCs were isolated from fresh blood using density gradient centrifugation (Histopaque, Sigma-Aldrich H889-500ML). For flow cytometry of surface markers, 250,000 PBMCs were stained with Live/death fixable viability stain, CD3-APC-Vio770, CD56 PerCP-Vio700, CD11B BV421, CD16 FITC, NKp46/CD335, NKG2D, and measured (antibodies and gating strategy: supplementary table 9 and Figure S4).

For intracellular staining, PBMCs were used for NK cell isolation using magnetic bead separation assays (Human NK isolation kit, Miltenyi 130-092-657). 50,000 NK cells per well were plated in 96 round-bottom plates. Cells were stained with viability stain, CD3-APC-Vio770, CD56 PerCP-Vio700, CD11B BV421. Hereafter, fixation and permeabilization solutions were added (Invitrogen 00-5123-43, 00-5223-56, 00-8333-56). After incubation, TNF-PE and IFNg-V450 antibodies were added and measured. Singlets were identified based on scatter plots. NK cells were identified as CD3^-^CD56^+^ cells from live cells. All experiments included isotype controls and auto-fluorescent controls.

For co-culturing experiments, human peripheral blood cells from a healthy donor were differentiated into human monocyte-derived macrophages as described (5). M2-polarized macrophages were obtained by incubation with 20 ng/ml IL-4 (Miltenyi 130-093-924) and 100 µg/mL IL-10 (Thermofisher PHC0105) for 16 hours. 50,000 isolated NK cells were added on confluent macrophages for 24h. Macrophages were harvested for flow cytometry analysis using fixable viability stain and CD64-BV421, CCR7-FITC, CD86-APC or CD206-BV421 and CD209-FITC (antibodies and gating strategies: supplementary table 10 and Figure S5).

*Plasma biomarkers of low-grade inflammation*

The assessment of biomarkers of low-grade inflammation (C-reactive protein, serum amyloid A, interleukin 6, interleukin 8, soluble intercellular adhesion molecule 1), and tumor necrosis factor) were assessed by a multiarray detection system based on electro-chemiluminescence technology as described previously (6).

*Quantification and statistical analysis*

For each analysis, the number of individuals, data expression, and statistical test used are indicated in each figure and table legend. Throughout the results section, data are expressed as mean ± SD or median [IQR]. Differences between groups were assessed by means of two-tailed independent Student’s t-tests or Mann-Whitney U tests where appropriate. We used Pearson’s correlation coefficients and linear regression analyses to investigate the associations between VAT, VAT NK cells, CD11B surface expression, z-scores of LGI, plasma TNF levels, z-scores of macrophage polarization, intracellular TNF levels, and insulin resistance where appropriate. Log transformation, square root transformation, or reciprocal transformation of variables were performed if warranted to not violate the assumptions of the statistical tests. Analyses were performed using SPSS version 20. Multiple mediation analyses were carried out with the PROGRESS plug-in for SPSS version 2.13 (A.F. Hayes, Ohio State University, OH, USA). All regression and mediation models were adjusted for potential confounders and, if warranted, specified per analysis.

In the co-incubation experiments, we tested whether NK cells from obese individuals, compared to those of lean individuals, induced an inflammatory polarization of macrophages. To test this hypothesis, we calculated an overall z-score to represent macrophage polarization. For its calculation, a z-score was first calculated for each individual M1 or M2 marker according to the formula: (individual value – population mean)/population standard deviation. Then, we negated the z-scores of the M2 markers CD209 and CD206 to ensure the same directionality as for the measured M1 markers. Finally, we calculated the average z-score for each individual (i.e. mean of –(zCD209), -(zCD206), zCCR7, zCD86, and zCD64), and calculated the overall polarization z-score. With this method, multiple testing is avoided, statistical efficiency is increased, and the influences of biological variability and measurement error are reduced. In the validation cohort, fully adjusted statistical models (i.e. adjustment for age, sex, glucose metabolism status, systolic blood pressure, total-to-HDL cholesterol ratio, smoking status, prior cardiovascular disease, and use of antihypertensive, anti-inflammatory, glucose-lowering or lipid-lowering medication) were reported, unless mentioned otherwise. Two-tailed p-values of < 0.05 were considered statistically significant.

1. Wouters K, Gaens K, Bijnen M, Verboven K, Jocken J, Wetzels S, et al. Circulating classical monocytes are associated with CD11c+ macrophages in human visceral adipose tissue. *Sci Rep.* 2017;7:42665.

2. Kusters YH, Schalkwijk CG, Houben AJ, Kooi ME, Lindeboom L, Op 't Roodt J, et al. Independent tissue contributors to obesity-associated insulin resistance. *JCI Insight.* 2017;2(13).

3. DeFronzo RA, Tobin JD, and Andres R. Glucose clamp technique: a method for quantifying insulin secretion and resistance. *Am J Physiol.* 1979;237(3):E214-23.

4. Schram MT, Sep SJS, van der Kallen CJ, Dagnelie PC, Koster A, Schaper N, et al. The Maastricht Study: an extensive phenotyping study on determinants of type 2 diabetes, its complications and its comorbidities. *Eur J Epidemiol.* 2014;29(6):439-51.

5. Fuentes L, Wouters K, Hannou SA, Cudejko C, Rigamonti E, Mayi TH, et al. Downregulation of the tumour suppressor p16INK4A contributes to the polarisation of human macrophages toward an adipose tissue macrophage (ATM)-like phenotype. *Diabetologia.* 2011;54(12):3150-6.

6. van Bussel BC, Ferreira I, van de Waarenburg MP, van Greevenbroek MM, van der Kallen CJ, Henry RM, et al. Multiple inflammatory biomarker detection in a prospective cohort study: a cross-validation between well-established single-biomarker techniques and an electrochemiluminescense-based multi-array platform. *PLoS One.* 2013;8(3):e58576.

**Supplementary figure 1**

*A. Representative flow cytometry stainings of MDMs either cultured without (orange) or with (green) NK cells from an obese individual. Control stainings are depicted in grey. B. Representative flow cytometry staining of intracellular TNF in blood NK cells from a lean (red) and obese (blue) individual. Control staining is depicted in grey. C. Representative flow cytometry staining of CD11B on the surface of blood NK cells from a lean (red) and obese (blue) individual. Control staining is depicted in grey.*

**Supplementary Figure 2: gating strategy blood NK cells**

From single blood cells, single and live cells were gated based on forward and side scatters. From live cells, negative (dump) selection was done based on CD3 (T cells), CD19 (B cells) and CD66b (granulocytes). Dump^-^ cells were gated for CD56 (NK cells).

**Supplementary Figure 3: gating strategy human adipose tissue**

SVF cells from vAT and scAT were gated for live single cells based on forward and side scatters. Immune cells were selected based on CD45 expression. Lymphocyte populations in AT were identified based on CD19 for B-cells and CD3 for T-cellsNon-B- and T-cells were gated for CD56 to identify NK cells. From CD45^+^ cells B-cells (CD19), T-cells (CD3), NK cells (CD56) and granulocytes (CD66b) were excluded for the identification of macrophages (defined as CD11Bhi), which were subdivided in CD11B^+^CD11C^-^ and CD11B^+^CD11C^+^ macrophages.

**Supplementary Figure 4: Gating strategy isolated blood NK cells**

Singlets were identified based on scatter plots. Live lymphocytes were gated using viability stain, after which NK cells were identified as CD3^-^CD56^+^ cells.

**Supplementary figure 5: Gating strategy primary human monocyte derived macrophages**

Live single cells were gated based on forward and side scatters. Live cells were gated using viability staining and surface expression of polarization markers was assessed as MFI.

| **Supplementary table 1. Biopsy study population characteristics** | | | |
| --- | --- | --- | --- |
|  | Lean  (n = 15) | Obese  (n = 17) | P-value |
| Age [Yr] | 52.1 ± 6.1 | 49.5 ± 5.8 | 0.215 |
| Weight [kg] | 76.0 [73.0 – 81.0] | 115.0 [111.2 – 133.8] | < 0.001 |
| Fat Free Mass [kg] | 58.2 ± 5.8 | 76.2 ± 7.9 | < 0.001 |
| Fat Mass [kg] | 16.2 [14.7 – 20.5] | 41.8 [36.9 – 48.9] | < 0.001 |
| BMI [kg/m^2^] | 23.6 [22.7 – 24.1] | 37.1 [35.1 – 38.7] | < 0.001 |
| Waist Circumference [cm] | 89.0 [86.0 – 93.0] | 124.0 [121.3 – 130.3] | < 0.001 |
| Hip Circumference [cm] ^a^ | 92.5 [89.5 – 94.5] | 114.8 [111.6 – 118.8] | < 0.001 |
| HOMA-IR | 1.73 [1.27 – 3.30] | 5.63 [3.54 – 7.16] | < 0.001 |
| Fasting Plasma Glucose [mmol/L] | 5.44 ± 0.93 | 6.08 ± 0.97 | 0.069 |
| HbA1c [%] | 5.2 [5.1 – 5.5] | 5.6 [5.5 – 6.1] | < 0.001 |
| HbA1c [mmol/mol] | 33 [32 – 37] | 38 [37 – 43] | < 0.001 |
| Diabetes Status [Y/N] | 0 / 15 | 3 / 14 | N/A |
| Glucose lowering Therapy [Y/N] | 0 / 15 | 3 / 14 | N/A |
| Systolic Blood Pressure [mmHg] ^b^ | 132.3 ± 15.1 | 145.6 ± 18.1 | 0.053 |
| Diastolic Blood Pressure [mmHg] ^b^ | 83.6 ± 9.2 | 83.8 ± 8.4 | 0.956 |
| White Blood Cell Count, Blood [x10^9/L] | 7.81 ± 2.27 | 6.63 ± 1.39 | 0.094 |
| Neutrophilic Granulocyte Count, Blood [x10^9/L] | 4.62 [3.61 – 6.18] | 3.70 [3.11 – 4.70] | 0.235 |
| Eosinophilic Granulocyte Count, Blood [x10^9/L] | 0.22 [0.20 – 0.38] | 0.15 [0.11 – 0.26] | 0.040 |
| Basophilic Granulocyte Count, Blood [x10^9/L] | 0.05 ± 0.02 | 0.04 ± 0.02 | 0.105 |
| Lymphocyte Count, Blood [x10^9/L] | 1.99 ± 0.50 | 1.78 ± 0.69 | 0.333 |
| Monocyte Count, Blood [x10^9/L] | 0.46 [0.37 – 0.69] | 0.54 [0.41 – 0.57] | 0.761 |
| NK Cell Count, Blood [x10^9/L] ^a^ | 0.20 [0.14 – 0.41] | 0.28 [0.15 – 0.44] | 0.401 |
| NK-CD11B MFI, Blood [AU] ^c^ | 4953 ± 1083 | 7126 ± 1342 | < 0.001 |
| SAT NK Cell Count [% of Live] ^d^ | 3.52 ± 2.10 | 3.77 ± 1.84 | 0.758 |
| VAT NK Cell Count [% of Live] ^e^ | 2.70 [1.64 – 4.09] | 3.79 [2.66 – 6.02] | 0.025 |
| VAT M1/M2 Ratio | 0.78 [0.62 – 1.09] | 1.05 [0.85 – 2.55] | 0.074 |
| VAT TNF Expression [Fold of Control] ^f^ | 0.66 [0.48 – 1.33] | 1.28 [0.71 – 2.17] | 0.062 |
| Data presented as mean ± SD or median [IQR]. Baseline differences between lean and obese men were assessed by means of independent Student’s T-test or Mann-Whitney U test, where appropriate. ^a^ Analyzed in 15 lean and 16 obese men; ^b^ analyzed in 11 lean and 17 obese men; ^c^ analyzed in 14 lean and 16 obese men; ^d^ analyzed in 12 lean and 14 obese men; ^e^ analyzed in 15 lean and 16 obese men; ^f^ analyzed in 15 lean and 16 obese men. | | | |

| **Supplementary table 2. Metabolic phenotyping study population characteristics** | | | |
| --- | --- | --- | --- |
|  | Lean  (n = 22) | Obese  (n = 42) | P-value |
|  |  |  |  |
| Age [Yr] | 55.0 [23.2 – 61.2] | 52.4 [46.5 – 61.4] | 0.661 |
| Weight [kg] | 75.8 ± 8.2 | 96.4 ± 8.8 | < 0.001 |
| Fat Free Mass [kg] | 62.8 ± 5.6 | 70.6 ± 6.0 | < 0.001 |
| Fat Mass [kg] | 13.0 ± 4.6 | 25.9 ± 4.8 | < 0.001 |
| BMI [kg/m^2^] | 23.4 ± 1.9 | 30.1 ± 2.0 | < 0.001 |
| Waist Circumference [cm] | 85.8 [80.6 – 89.8] | 106.6 [103.5 – 109.1] | < 0.001 |
| Hip Circumference [cm] | 96.8 ± 4.4 | 107.3 ± 5.5 | < 0.001 |
| Visceral Adipose Tissue Volume [L] ^a^ | 0.872 ± 0.434 | 2.384 ± 0.751 | < 0.001 |
| Subcutaneous Adipose Tissue Volume [L] ^a^ | 1.437 ± 0.527 | 3.067 ± 0.798 | < 0.001 |
| Whole-Body Glucose Disposal [mg/kg/min] | 6.85 ± 1.85 | 4.04 ± 1.23 | < 0.001 |
| HOMA-IR | 1.7 ± 0.5 | 2.9 ± 1.3 | < 0.001 |
| Fasting Plasma Glucose [mmol/L] | 5.36 ± 0.28 | 5.66 ± 0.46 | 0.002 |
| HbA1c [%] ^b^ | 5.15 ± 0.38 | 5.28 ± 0.39 | 0.211 |
| HbA1c [mmol/mol] ^b^ | 32.7 ± 4.2 | 34.1 ± 4.2 | 0.211 |
| Systolic Blood Pressure [mmHg] | 116.5 ± 8.1 | 122.1 ± 8.4 | 0.012 |
| Diastolic Blood Pressure [mmHg] | 71.1 ± 8.6 | 79.7 ± 6.9 | < 0.001 |
| White Blood Cell Count, Blood [x10^9/L] ^c^ | 4.89 ± 1.32 | 5.67 ± 1.25 | 0.027 |
| Neutrophilic Granulocyte Count, Blood [x10^9/L] ^d^ | 2.59 ± 0.88 | 3.16 ± 1.01 | 0.032 |
| Eosinophilic Granulocyte Count, Blood [x10^9/L] ^d^ | 0.22 ± 0.13 | 0.21 ± 0.13 | 0.789 |
| Basophilic Granulocyte Count, Blood [x10^9/L] ^d^ | 0.03 [0.00 – 0.05] | 0.03 [0.00 – 0.06] | 0.894 |
| Lymphocyte Count, Blood [x10^9/L] ^d^ | 1.60 ± 0.37 | 1.77 ± 0.46 | 0.151 |
| Monocyte Count, Blood [x10^9/L] ^d^ | 0.46 ± 0.14 | 0.52 ± 0.15 | 0.128 |
| NK Cell Count, Blood [x10^9/L] ^d^ | 0.10 [0.08 – 0.15] | 0.14 [0.10 – 0.19] | 0.037 |
| NK-CD11B MFI, Blood [AU] | 10999 ± 2445 | 12865 ± 2664 | 0.008 |
| Low-grade Inflammation Z-score | -0.21 ± 0.73 | 0.11 ± 0.60 | 0.059 |
| Plasma TNF [pg/mL] | 1.85 [1.50 – 2.05] | 1.96 [1.62 – 2.31] | 0.161 |
| Plasma IL-6 [pg/mL] | 0.58 [0.32 – 0.97] | 0.64 [0.47 – 0.90] | 0.344 |
| Data presented as mean ± SD or median [IQR]. Baseline differences between lean and obese men were assessed by means of independent Student’s T-test or Mann-Whitney U test where appropriate. ^a^ Analyzed in 21 lean and 42 obese men; ^b^ analyzed in 22 lean and 41 obese men; ^c^ analyzed in 21 lean and 39 obese men; ^d^ analyzed in 21 control and 38 obese men. | | | |

| **Supplementary Table 3. Validation study population characteristics – The Maastricht Study** | | | | |
| --- | --- | --- | --- | --- |
|  | NGT  (n = 480) | Prediabetes  (n = 109) | T2DM  (n = 250) | P-value |
|  |  |  |  |  |
| Age [Yr] | 58.5 ± 8.5 | 60.3 ± 8.5 ^***^ | 63.7 ± 8.2 ^###^ | < 0.001 |
| Sex [M/F] | 199/281 | 49/60 | 160/90 ^###^ | < 0.001 |
| Smoking Status [never/former/current] | 207/218/55 | 35/56/18 | 78/130/42 ^##^ | 0.010 |
| Weight [kg] | 74.1 ± 13.2 | 78.1 ± 12.9 ^**^ | 87.5 ± 15.9 ^###^ | < 0.001 |
| Fat Free Mass [kg] | 47.4 [40.9 – 57.7] | 49.7 [41.3 – 57.0] | 55.8 [46.6 – 62.3] ^###^ | < 0.001 |
| Fat Mass [kg] | 23.9 [20.0 – 28.8] | 26.8 [22.0 – 34.8] ^***^ | 30.9 [24.4 – 39.4] ^###^ | < 0.001 |
| BMI [kg/m^2^] | 25.5 ± 3.5 | 27.5 ± 4.4 ^***^ | 29.9 ± 5.1 ^###^ | < 0.001 |
| Waist Circumference [cm] | 89.4 ± 10.9 | 96.7 ± 11.2 ^***^ | 105.1 ± 13.1 ^###^ | < 0.001 |
| Hip Circumference [cm] | 99.2 [95.0 – 104.0] | 100.5 [95.8 – 107.1] ^**^ | 103.6 [99.0 – 111.3] ^###^ | < 0.001 |
| Visceral Adipose Tissue [cm^2^] ^a^ | 112.8 [69.4 – 172.9] | 160.8 [117.0 – 220.7] ^***^ | 248.5 [171.0 – 317.3] ^###^ | < 0.001 |
| Fasting Plasma Glucose [mmol/L] | 5.20 ± 0.41 | 5.85 ± 0.61 ^***^ | 7.61 ± 1.51 ^###^ | < 0.001 |
| HOMA-IR ^b^ | 1.20 [0.88 – 1.65] | 1.71 [1.21 – 2.70] ^***^ | 2.27 [1.30 – 3.19] ^###^ | < 0.001 |
| Matsuda Index ^c^ | 4.27 [3.10 – 6.18] | 2.07 [1.34 – 3.44] ^***^ | 1.79 [1.10 – 2.97] ^###^ | < 0.001 |
| HbA1c [%] | 5.3 [5.1 – 5.5] | 5.6 [5.3 – 5.9] ^***^ | 6.6 [6.1 – 7.0] ^###^ | < 0.001 |
| HbA1c [mmol/mol] | 35 [32 – 37] | 37 [34 – 41] ^***^ | 48 [44 – 53] ^###^ | < 0.001 |
| Systolic Blood Pressure [mmHg] | 130.2 ± 17.0 | 133.8 ± 16.0 ^*^ | 138.4 ± 16.9 ^###^ | < 0.001 |
| Diastolic Blood Pressure [mmHg] | 77.4 ± 9.9 | 76.3 ± 9.6 | 75.5 ± 9.5 | 0.125 |
| Glucose lowering Therapy [%] | 0 | 0 | 75.6 | N/A |
| Blood Pressure Lowering Therapy [%] | 20.8 | 44.0 ^***^ | 69.2 ^###^ | < 0.001 |
| Lipid Lowering Therapy [%] | 14.2 | 39.4 ^***^ | 72.0 ^###^ | < 0.001 |
| Anti-inflammatory Therapy [%] | 12.7 | 16.5 | 13.2 | 0.572 |
| History of Cardiovascular Disease [%] | 11.9 | 17.4 | 25.6 ^###^ | < 0.001 |
| White Blood Cell Count, Blood [x10^9/L] | 5.05 [4.30 – 5.90] | 5.60 [4.70 – 6.80] ^***^ | 6.00 [5.10 – 7.30] ^###^ | < 0.001 |
| Neutrophilic Granulocyte Count, Blood [x10^9/L] | 2.76 [2.24 – 3.35] | 3.23 [2.47 – 4.14] ^***^ | 3.46 [2.85 – 4.19] ^###^ | < 0.001 |
| Eosinophilic Granulocyte Count, Blood [x10^9/L] | 0.14 [0.09 – 0.20] | 0.17 [0.11 – 0.22] ^*^ | 0.17 [0.11 – 0.24] ^###^ | < 0.001 |
| Lymphocyte Count, Blood [x10^9/L] | 1.63 [1.37 – 1.98] | 1.72 [1.43 – 2.07] | 1.84 [1.52 – 2.24] ^###^ | < 0.001 |
| Monocyte Count, Blood [x10^9/L] | 0.42 [0.34 – 0.50] | 0.46 [0.36 – 0.58] ^***^ | 0.48 [0.40 – 0.61] ^###^ | < 0.001 |
| NK Cell Count, Blood [x10^9/L] | 0.19 [0.13 – 0.27] | 0.21 [0.14 – 0.27] | 0.22 [0.14 – 0.31] ^#^ | 0.121 |
| NK-CD11B MFI, Blood [AU] | 8866 ± 2188 | 9140 ± 2200 | 9419 ± 2291 ^###^ | 0.006 |
| Low-grade Inflammation Z-score | -0.34 ± 0.92 | 0.27 ± 0.94 ^***^ | 0.53 ± 0.91 ^###^ | < 0.001 |
| Plasma TNF [pg/mL] | 2.06 [1.75 – 2.40] | 2.17 [1.93 – 2.62] ^***^ | 2.46 [2.15 – 2.82] ^###^ | < 0.001 |
| Plasma IL-6 [pg/mL] | 0.47 [0.30 – 0.71] | 0.59 [0.42 – 0.90] ^***^ | 0.81 [0.56 – 1.10] ^###^ | < 0.001 |
| Data presented as mean ± SD or median [IQR]. Baseline differences between individuals with prediabetes and normal glucose metabolism (NGM), and type 2 diabetes (T2DM) and NGT were assessed by means of independent samples Student’s T-test, Mann-Whitney U test, or chi-square test where appropriate. P-value prediabetes compared to NGM; * = p < 0.05, ** = p < 0.01, *** = p < 0.001. P-value T2DM compared to NGM; ^#^ = p < 0.05, ^##^ = p < 0.01, ^###^ = p < 0.001. Differences between all groups were assessed by means of one-way ANOVA, Kruskal-Wallis test, or chi-square test where appropriate; respective p-values reported in separate column. ^a^ Analyzed in 405 NGM, 93 prediabetes, and 198 T2DM; ^b^ analyzed in 310 NGM, 73 prediabetes, and 179 T2DM; ^c^ analyzed in 313 NGM, 71 prediabetes, and 156 T2DM. | | | | |

| **Supplementary table 4 – Associations between VAT and NK-CD11B (n = 696)** | | | | |
| --- | --- | --- | --- | --- |
|  | Model | NK-CD11B [AU] | | |
|  |  | β (95%-CI) | p-value | |
|  |  |  |  |  |
| VAT [cm^2^] | 1 | 3.90 (1.84; 5.97) | < 0.001 |  |
|  | 2 | 3.21 (0.94; 5.48) | 0.006 |  |
|  |  |  |  |  |
|  | | | | |
| Model 1: Adjusted for age, sex, and glucose metabolism status. Model 2: Fully adjusted model (model 1 + systolic blood pressure, total-to-HDL cholesterol ratio, smoking status, prior cardiovascular disease, and use of antihypertensive, anti-inflammatory, glucose-lowering or lipid-lowering medication). Multiple linear regression analyses in individuals with normal glucose metabolism (n = 405), prediabetes (n = 93), and type 2 diabetes (n = 198). The beta of each model is presented along with its respective 95%-CI and p-value. | | | | |

| **Supplementary Table 5 – Associations between NK-CD11B and z-LGI, plasma TNF or plasma IL-6 (n = 839)** | | | | |
| --- | --- | --- | --- | --- |
|  | Model | z-LGI | | |
|  |  | β (95%-CI) | p-value | |
|  |  |  |  |  |
| NK-CD11B (x1000) | 1 | 0.039 (0.010; 0.067) | 0.007 | |
|  | 2 | 0.028 (0.000; 0.055) | 0.048 | |
|  |  |  |  | |
|  | Model | z-TNF | | |
|  |  | β (95%-CI) | p-value | |
|  |  |  |  | |
| NK-CD11B (x1000) | 1 | 0.040 (0.011; 0.070) | 0.007 | |
|  | 2 | 0.034 (0.005; 0.064) | 0.023 | |
|  |  |  |  | |
|  | Model | z-IL-6 | | |
|  |  | β (95%-CI) | p-value | |
|  |  |  |  | |
| NK-CD11B (x1000) | 1 | 0.073 (0.044; 0.102) | < 0.001 | |
|  | 2 | 0.042 (0.014; 0.071) | 0.004 | |
|  |  |  |  | |
|  | | | | |
| Model 1: Adjusted for age, sex, and glucose metabolism status. Model 2: Fully adjusted model (model 1 + systolic blood pressure, total-to-HDL cholesterol ratio, smoking status, prior cardiovascular disease, and use of antihypertensive, anti-inflammatory, glucose-lowering or lipid-lowering medication). Multiple linear regression analyses in individuals with normal glucose metabolism (n = 480), prediabetes (n = 109), and type 2 diabetes (n = 250). The beta of each model is presented along with its respective 95%-CI and p-value. | | | | |

| **Supplementary table 6 – Associations between NK-CD11B and the Matsuda Index (n = 540)** | | | | |
| --- | --- | --- | --- | --- |
|  | Model | Matsuda Index | | |
|  |  | β (95%-CI) | p-value | |
|  |  |  |  |  |
| NK-CD11B | 1 | 2337 (609; 4064) | 0.008 |  |
|  | 2 | 2212 (440; 3783) | 0.013 |  |
|  |  |  |  |  |
|  | | | | |
| Model 1: Adjusted for age, sex, and glucose metabolism status. Model 2: Fully adjusted model (model 1 + systolic blood pressure, total-to-HDL cholesterol ratio, smoking status, prior cardiovascular disease, and use of antihypertensive, anti-inflammatory, glucose-lowering or lipid-lowering medication). Multiple linear regression analyses in individuals with normal glucose metabolism (n = 313), prediabetes (n = 71), and type 2 diabetes (n = 156). The beta of each model is presented along with its respective 95%-CI and p-value. NK-CD11B was transformed to 1 / NK-CD11B and the Matsuda Index to SQRT Matsuda Index to fulfill the assumptions of the regression model and are presented as such. | | | | |

**Supplementary table 7. Antibodies used for flow cytometry of blood cells**

| **Marker** | **fluorochrome** | **clone** | **manufacturer** |
| --- | --- | --- | --- |
| CD3  CD19  CD66B | FITC | UCHT1  HIB19  G10F5 | BD  BD  BD |
| CD56 | PE | MY31 | BD |
| HLA-DR | V500 | G46-6 | BD |
| CD14 | APC-H7 | MφP9 | BD |
| CD16 | PerCP | 3G8 | BD |
| CD11B | BV421 | ICRF44 | BD |
| CD11C | PE-Cy7 | B-ly6 | BD |
| CX3CR1 | APC | 2A9-1 | BioLegend |

**Supplementary table 8. Antibodies used for flow cytometry of SVF cells**

| **Marker** | **fluorochrome** | **clone** | **manufacturer** |
| --- | --- | --- | --- |
| **Cocktail 1** |  |  |  |
| CD3  CD19  CD66B  CD56 | FITC | UCHT1  HIB19  G10F5  B159 | BD  BD  BD  BD |
| CD11C | APC-H7 | Bu15 | BioLegend |
| CD11B | BV421 | ICRF44 | BD |
| CD45 | PE-Cy7 | HI30 | BD |
| **Cocktail 2** |  |  |  |
| CD45 | PE-Cy7 | HI30 | BD |
| CD4 | PerCP | RPA-T4 | BioLegend |
| CD8 | APC-H7 | SK1 | BD |
| CD56 | APC | HCD56 (NCAM) | BioLegend |
| CD19 | BV421 | HIB19 | BioLegend |
| CD3 | V500 | UCHT1 | BD |

**Supplementary table 9. Antibodies used for flow cytometry of isolated NK cells**

| **Marker** | **fluorochrome** | **clone** | **manufacturer** |
| --- | --- | --- | --- |
| CD56 | PerCP-Vio700 | REA 196 | Miltenyi |
| CD3 | APC-Vio770 | BW264/56 | Miltenyi |
| CD11B | BV421 | ICRF44 | BD |
| TNF | PE | Mab 11 | BioLegend |
| Viability | V510 |  | BD |

**Supplementary table 10. Antibodies used for flow cytometry of primary human macrophages**

| **Marker** | **fluorochrome** | **clone** | **manufacturer** |
| --- | --- | --- | --- |
| CD64 | BV421 | 10.1 | BioLegend |
| CCR7 | FITC | G043H7 | BioLegend |
| CD86 | APC | IT2.2.2 | BioLegend |
| CD206 | BV421 | 19.2 | BD |
| CD209 | FITC | 9E9A8 | BioLegend |
| Viability | V510 |  | BD |
